# Characterization of the salivary microbiome before and after antibiotic therapy via separation technique

**DOI:** 10.1007/s00253-023-12371-0

**Published:** 2023-02-27

**Authors:** Katarzyna Pauter-Iwicka, Viorica Railean, Michał Złoch, Paweł Pomastowski, Małgorzata Szultka-Młyńska, Dominika Błońska, Wojciech Kupczyk, Bogusław Buszewski

**Affiliations:** 1 Department of Environmental Chemistry and Bioanalytics, Faculty of Chemistry, Nicolaus Copernicus University, Gagarina 7, 87-100 Torun, Poland; 2 Centre for Modern Interdisciplinary Technologies, Nicolaus Copernicus University, Wilenska 4, 87-100 Torun, Poland; 3 Department of Infectious, Invasive Diseases and Veterinary Administration, Institute of Veterinary Medicine, Nicolaus Copernicus University in Torun, Gagarina 7, 87-100 Toruń, Poland; 4 Department of General, Gastroenterological&Oncological Surgery Collegium Medicum, Nicolaus Copernicus University, Torun, Poland

**Keywords:** Antibiotics, Therapy, Salivary bacteria, Microbiota, Mass spectrometry, Proteomics

## Abstract

**Abstract:**

In the present research, the MALDI-TOF MS technique was applied as a tool to rapidly identify the salivary microbiome. In this fact, it has been monitored the changes occurred in molecular profiles under different antibiotic therapy. Significant changes in the composition of the salivary microbiota were noticed not only in relation to the non antibiotic (non-AT) and antibiotic treatment (AT) groups, but also to the used media, the antibiotic therapy and co-existed microbiota. Each antibiotic generates specific changes in molecular profiles. The highest number of bacterial species was isolated in the universal culture medium (72%) followed by the selective medium (48% and 38%). In the case of non-AT patients, the prevalence of *Streptococcus salivarius* (25%), *Streptococcus vestibularis* (19%), *Streptococcus oralis* (13%), and *Staphylococcus aureus* (6%) was identified while in the case of AT, *Streptococcus salivarius* (11%)*, Streptococcus parasanguinis* (11%), *Staphylococcus epidermidis* (12%), *Enterococcus faecalis* (9%), *Staphylococcus hominis* (8%), and *Candida albicans* (6%) were identified. Notable to specified that the *Candida albicans* was noticed only in AT samples, indicating a negative impact on the antibiotic therapy.

The accuracy of the MALDI-TOF MS technique was performed by the 16S rRNA gene sequencing analysis—as a reference method. Conclusively, such an approach highlighted in the present study can help in developing the methods enabling a faster diagnosis of disease changes at the cellular level before clinical changes occur. Once the MALDI tool allows for the distinguishing of the microbiota of non-AT and AT, it may enable to monitor the diseases treatment and develop a treatment regimen for individual patients in relation to each antibiotic.

**Key points:**

- *The salivary microbiota of antibiotic-treated patients was more bacteria variety*
- *MALDI-TOF MS is a promising tool for recording of reproducible molecular profiles*
- *Our data can allow to monitor the treatment of bacterial diseases for patients*

## Introduction

Since the time when the first antibiotics were introduced to treat bacterial infections, drug resistance to pathogens has become a serious health problem. The reasons include various factors such as an irresponsible dosage of antibiotics, naturally occurring mutations, and the transmission of drug-resistant strains. Microorganisms can have both positive and negative impacts. Many of them can make food go bad and cause serious diseases. For this reason, it is extremely important to search for quick and reliable methods to identify the basic infectious agents such as bacteria, which is particularly important in the medical diagnostics (Jackowski et al. 2008; Pauter et al. 2020).

Personalized treatments are one of the most important achievements of modern medicine (Garzón et al. 2020). For this field to develop it is necessary for specialists in the field of biology, genetics, biotechnology, bioinformatics, and pharmacology to cooperate with the medical community. This leads to an innovative approach in the diagnostics and, in consequence, in the medical treatment by improving or adapting the pharmacological therapy to the individual needs of patients, the so-called targeted pharmacological therapy, “tailor-made therapy” or personalized medicine (Borg-Bartolo et al. 2020).

On the other hand, the diversity and composition of saliva microbiota seem highly important for the human health and disease. Hence, in the recent years, saliva has attracted widespread interest as a means of simple and rapid testing because the composition of it might reflect the health status. The quick identification of the pathogen causing the infection will enable the implementation of an appropriate therapy (Jackowski et al. 2008; Pauter et al. 2020). Currently, the MALDI-TOF MS is used with great success (Złoch et al. 2020b). The worthwhile point is that this technique is often chosen in the identification of microorganisms for routine clinical testing (Hou et al. 2019; Duncan and DeMarco 2019; Van Belkum et al. 2017).

The human oral microbiome is one of the most active environments for many species of bacteria, where they undergo an extensive interspecies competition to form a multispecies biofilm structure. These bacteria are also present in saliva; they constitute many hundreds and thousands of species, some of which are unique to this specific habitat (Gao et al. 2018). *Streptococcus salivarius* is considered to be the first human oral colonizer at birth and can therefore play a role in setting up immune homeostasis and controlling the inflammatory reactions of the host. *Streptococcus mitis*, *Streptococcus oralis*, and *Streptococcus anginosus* prefer to colonize on oral soft tissues and saliva, while *Streptococcus sanguinis* tends to colonize on teeth (Abranches et al. 2018). There are also opportunistic species among *Streptococcus* bacteria like *Streptococcus mutants*. Its contribution to caries development is well established (El-sherbiny 2014;Koo and Bowen 2014). Moreover, various *Lactobacillus* species, especially *L. fermentum*, *L. rhamnosus*, *L. salivarius*, *L. casei*, *L. acidophilus*, and *L. plantarum* are frequent mouth inhabitants and studies show that they antagonize harmful microorganisms and reinforce the dental health (Koll-Klais et al. 2005; Badet and Thebaud 2008; Wasfi et al. 2018).

The other group of microbes in the oral cavity includes *Candida* species, especially during a long-term antibiotic therapy (Muzyka and Glick 1995). In many individuals, *C. albicans* is a minor component of their oral flora, which does not generate any clinical symptoms (Cannon and Chaffin 1999). In contrast, when the balance of the microbiota in the oral cavity is disturbed, candida seeks to colonize the oral tissue by creating a biofilm with *Streptococcus*, which plays a pathogenic role (Tsui et al. 2016; Koo et al. 2018).

According to the recent medical reports and current scientific knowledge, a change in the balance of the oral bacterial composition has the potential to signal pathological conditions. This includes diseases such as halitosis (Haraszthy et al. 2007), caries (Guo and Shi 2013), and periodontosis (Ko et al. 2020), but also systemic diseases including breathing diseases (Gomes-Filho et al. 2010), diabetics (Sabharwal et al. 2019) along with cardiovascular diseases (Fernandes et al. 2014), and cancer (Mager et al. 2005). The characteristics of the salivary microbiome in obese subjects also received attention (Al-Rawi and Al-Marzooq 2017). Nevertheless, healthy salivary bacterium should be identified primarily to describe the changes caused by the disease, which may eventually lead to the development of diagnostic tools to improve the treatment or prevent the disease (Espuela-Ortiz et al. 2019). Additionally, these several studies indicate that salivary bacteria biomarkers in the oral cavity constitute a recognized diagnostic and prognostic tool for a variety of diseases. Hence, many activities were undertaken using hyphenated methods based on the bacterial ribosomal proteins determination (MALDI-TOF MS) (Stîngu et al. 2008; Sun et al. 2016) along with volatile organic compounds (VOCs) detection (gas chromatography-mass spectrometry, GC-MS) (Milanowski et al. 2019). Nevertheless, most of the research on oral microbes utilize the 16SrRNA-based technique (Hrynkiewicz et al. 2008).

The literature often focuses on pathogenic microorganisms and the assessment of their significance in the etiology and course of infectious diseases along with the spread of drug resistance to commonly used antibacterial drugs. However, what is interesting is the fact whether and what differences in the prevalence of the bacterial strain colonization occur in people with bacterial infections undergoing the antibiotic therapy compared to non-antibiotic therapy. Hence, in this study, the salivary microbiota after antibiotic treatment was described. The MALDI-TOF MS technique as a tool to provide a rapid diagnosis and identification of microbiota was used; different media were investigated in order to achieve a complimentary microbiota identification. At the same time, we utilized the 16S rRNA gene sequencing to determine the selected salivary bacteria in order to obtain information on the effectiveness and accuracy of the investigated spectrometric method. Additionally, the present research focused on checking the ability of the MALDI technique for the investigation of fast monitoring of the patients under antibiotic therapy. The samples from 14 patients not treated with antibiotics were intended to determine possible changes in the local population related to both local epidemiological factors and hospitalization factors. This study was performed to determine the possible impact of the hospital environment on changes in the patient’s microbiome after 10 days of hospitalization with or without antibiotic treatment. Therefore, in the population of patients subjected to antibiotic therapy, the focus was on changing the above-mentioned profile, which allowed to separate the changes resulting from the hospitalization itself from those caused by antibiotic therapy. As we have presented in the profile of patients undergoing antibiotic therapy, we have noticed an increase in the diversity of strains; the emergence of bacteria typically associated with the surgical ward. The interesting point also was to compare if the MALDI technique would differentiate each administrated antibiotic in the relevant time period. Moreover, the optimal conditions of the growth medium for the identification of microorganism by using the MALDI-TOF MS were examined. A correlation between protein profile changes of the non-AT and AT microbiota was performed and studied in details. Additionally, the impact of antibiotic and pathogen’s presence on the patients’ therapy was described.

## Materials and methods

### Saliva samples preparation protocol

In this study, 38 samples were investigated; the saliva samples were provided from fourteen non-AT and twenty-four AT patients, who were hospitalized in very serious condition. The present research involved demographic data from 38 consecutive patients admitted to the Department of General, Gastroenterological and Oncological Surgery of the Nicolaus Copernicus University in Toruń. Patient data is strictly identified and marked with both name and surname, 11-digit ID number, description of the medical history number, date of sampling and type of antibiotic, its dose, and disease being the reason for its recommendation. Patients with diseases in the oral cavity were not eligible for the study. In some of the qualified patients, there were no indications for antibiotic therapy. This group was used to determine the local status of the salivary microbiome dependent on both population and hospitalization effects as mentioned. Initially, 42 patients were qualified for the study in the proportion of 14 non-antibiotic vs 28 antibiotic/2: 1 ratio; however, due to difficulties in obtaining the appropriate sample volume, 2 patients treated with an antibiotic were disqualified from the experiment—which was also a limitation of the study. Among the patients receiving antibiotic therapy, the participants undergoing the diabetic foot, surgical wounds, sinusitis, and phlegmon have been immediately subjected to the antibiotic therapy: azithromycin, amoxicillin, ciprofloxacin, clindamycin, cefotaxime and levofloxacin, metronidazole, and piperacillin. Non-antibiotic therapy patients were selected as a control. The average age of the subjects was 58.9 years old. Of these participants, 68% were men and the rest 32% were women. The present research did not classified the patients to the specific patient data (age, sex) once we were restricted in the sample number collection. Generally, all the patients were instructed to avoid eating, drinking, and brushing their teeth for 2 h before the saliva collection.

For the sample cultivation, an universal growth medium—*Brain Heart Infusion* (BHI) (Sigma-Aldrich, Germany) and two selective mediums such as the *Vancomycine Resistant Enterococci Agar Base* (VRE) (Sigma-Aldrich, Germany), and the *Azide Blood Agar BASE* (AZB) (Sigma-Aldrich, Germany) were chosen. The saliva was diluted with sterile peptone water (Sigma-Aldrich, Germany) in a 1:9 (v/v) ratio. The cultivation was performed by the serial dilution method based on the procedure of Abouassi and co-workers (Abouassi et al. 2014) with a slight modification. The peptone water was used instead of 0.9% NaCl to support the growth of the fastidious microorganisms. Subsequently, 100 μL of each suspension was streaked on the Petri dishes containing the chosen culture medium. Thereafter, they were incubated for 24 h (BHI, AZB) or 48 h (VRE) at a constant temperature of 37 °C in aerobic conditions. Moreover, the number of colony-forming units (CFU/mL) was determined by the colony counter (IUL S.A., Barcelona, Spain) and compared in non-AT and AT patients.

### Identification of salivary bacterial microbiome

#### MALDI-TOF MS measurements

All fresh colonies isolated in the different medium (as described in “Saliva samples preparation protocol” section) were then used for the identification. Owing to problems with the identification by the MALDI-TOF MS on AZB (considered as a selective medium), the respective medium was changed to the Tryptic Soy Agar (TSA, Sigma-Aldrich, Germany). TSA is considered a universal medium and applied as a routine diagnostic medium. The colonies isolated on the AZB medium were transferred to the TSA medium, incubated for 24 h at 37 °C in aerobic conditions, then identified using the MALDI tool.

The standard extraction protocol was adopted from our previous study, Pauter et al. with some changes (Pauter et al. 2022). The modification included suspending the pellet in 150 μl of distilled water and adding 450 μl of ethanol. Afterwards, the pellet was centrifuged for 5 min at 20 °C, 14, 400 rpm, then the supernatant was removed. Subsequently, the vacuum concentration was used to dry the pellet (8–10 min). The 70% formic acid (Merck, 98–100%, Germany), acetonitrile (Fluka Analytical Sigma Aldrich, Germany), was added into the dried pellet (1,1), and then centrifuged (2 min, 20 °C, 13, 000 rpm). Next, 1μl of the material was dropped into the MALDI target, left to dry and covered with 1 μl matrix alpha-cyano-4-hydroxycinnamic acid (HCCA) (Sigma-Aldrich, Switzerland). The external calibration of the instrument was performed using the Bacterial Test Standard, (BTS, Bruker, Bremen, Germany). Each spot was analyzed in duplicate in order to minimize the effect of changes in the sample preparation. The MALDI-TOF mass spectra measurements were carried out by an Ultraflextreme instrument (Bruker Daltonik, Bremen, Germany) operated in the positive ion mode using the BrukerBiotyper 1.1 software (Bruker Daltonik GmbH). The Flex Analysis 2.4 software (Bruker Daltonik GmbH, Bremen, Germany) was used to visualize the MS spectra. Moreover, the data were analyzed automatically by the MBT Compass software (Bruker Daltonik GmbH, Bremen, Germany) and the mass spectra were compared with the spectra of known microbial isolates from commercial libraries provided by Bruker Daltonik. Based on this data, the phyloproteomic tree (dendrogram) was prepared. The spectra match was evaluated by a proprietary algorithm and generated a logarithmic value (score) ranging from 0.0 to 3.0.

### Statistical analysis

The heat maps, the hierarchical clustering analysis, and radar chat were generated using the STATISTICAL Release version 7.0 software and Microsoft Excel 2010. All raw data were taken into consideration and correlated on the basis of the used medium and antibiotic, while the identified microbiota was performed.

### 16S rRNA gene sequencing

To correlate the data obtained from the identification by the MALDI-TOF MS, the 16 rRNA sequencing method was performed. There was an attempt to select one species (*Neisseria perflava*, *Enterococcus faecalis*, *Staphylococcus aureus*, *Streptococcus epidermidis*, *Streptococcus salivarius*, *Streptococcus pneumoniae*, *Staphylococcus cohnii*, and *Lactobacillus plantarum*) each of the identified microbial genus. Moreover, *Bacillus subtilis* was also chosen, based on the unreliable low score (1.6) generated by the MALDI-TOF MS.

The procedure of the DNA isolation was carried out according to the protocol supplied in the DNeasyUltraClean Microbial Kit (QIAGEN, Wrocław, Poland). The polymerase chain reaction amplification was performed using universal primers as forward: 27F(5-AGAGTTTGATCMTGGCTCAG-3) and reverse primers: 1492R(5-GGTTACCTTGTTACGACTT-3). After that, the PCR amplification products were purified and the sequencing of the amplified fragments was performed by using the Sanger dideoxy method by Genomed (Warsaw, Poland). Then, from the received sequences, the contigs were submitted using the BioEdit Sequence Alignment Editor ver. 7.2.5 software (12.11.2013) (Hall 1999). Finally, the Basic Local Alignment Search Tool (BLAST) database, available in the National Center for Biotechnology Information (NCBI) was used to compare the consensus sequences with the known 16S rRNA gene sequences deposited in the GenBank. The accession numbers of the studied DNA sequences were determined.

### Ethical considerations

This study was conducted according to the guidelines of the Declaration of Helsinki and approved by the Bioethical Commission of Collegium Medicum in Bydgoszcz of Nicolaus Copernicus University in Torun, Poland, according to the agreement number 477/2021 – 14.09.2021. A written informed consent was obtained from all the participants.

## Results

In the present research, the emphasis is put on the microbiota differences in patients with non- and under-antibiotic therapy. Moreover, to date, no work has been focused on the investigation of protein profile changes of isolate species identified in the patient’s microbiota not undergoing and undergoing the antibiotic therapy.

**Fig. 1:**
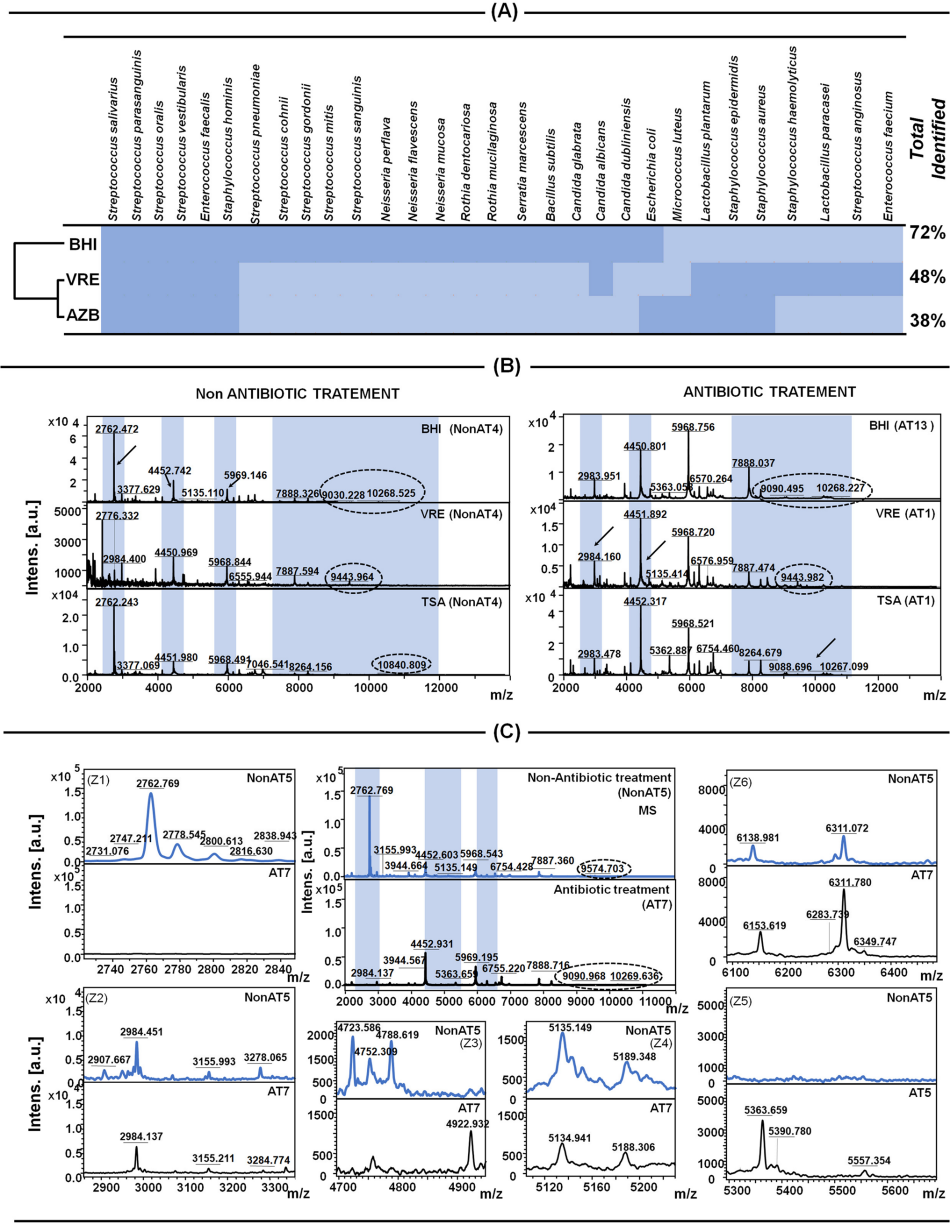
The abundance of isolated microorganisms in correlation with the used growth media (**A**) and the comparison between both non-AT as well as AT of the *S. salivarius* protein profiles in dependence of used culture media (**B**) and comparison between non-AT and AT patients on *S. vestibularis*, example – Z1–Z6 representing common and characteristic MS signals of each (**C**)

The colony-forming unit results indicate that in the saliva samples collected from the AT group bacteria cells were found between 10^5^ and 10^7^ CFU/mL. However, the number of CFU in the non-AT patients was noticed more than 10^7^ CFU/mL (64% of patients). Only in the case of non-AT2, non-AT6, non-AT7, non-AT8, and non-AT10, (36%) the bacterial count was around 10^6^ CFU/mL. The slight differences in the abundance can be associated with various lifestyles and distinct genotypes of the hosts (Ling et al. 2013). Figure 1A presents, the heat map combined with the dendrogram representing the total of isolated bacteria (%) in both group of patients (AT and non-AT) and differences between medium to isolate possible microbiota. The heat map was created to show the complimentary of the culture medium applied. The most percentage of isolated bacteria were observed in the universal growing media (BHI), which reported 72%, followed by the VRE medium (48%), and AZB medium (38%). Figure 1A shows that *Streptococcus salivarius*, *Streptococcus parasanguinis*, *Streptococcus oralis* and *Streptococcus vestibularis*, *Enterococcus faecalis*, *Staphylococcus hominis* were identified in all the cultivation medium.

Information regarding isolates identified in all the samples by the MALDI-TOF MS was presented in details (name of strain, score, and antibiotic used) in Table 1 (non-AT) and Table 2 (AT). Additionally, the used culture medium was also included. According to Tables 1 and 2, it can be observed that the log (score) value (level of identification) was in most cases above 2.0.

**Table 1:** Identification results of all isolates identified in non-AT patients using MALDI-TOF MS

Patient name	Antibotic	Best much in MALDI Biotyper database	Score value
Non-antibiotic treatment (non AT)			
Non-AT1	-	*Streptococcus salivarius 0807M25049501 IBS (BHI)*	2.06
		Streptococcus pneumoniae DSM 11868 DSM (BHI)*	2.11
		*Streptococcus parasanguinis DSM 6778T DSM_2 (VRE)*	1.85
Non-AT2	-	*Streptococcus vestibularis DSM 5636T DSM (BHI/VRE/TSA)*	2.11
		*Streptococcus sanguinis CCUG 29269 CCUG_corr (BHI)*	2.35
Non-AT3	-	*Streptococcus salivarius 0807M25049501 IBS (BHI)*	2.34
		*Streptococcus mitis V17_201158 MUZ (BHI)*	2.05
		*Streptococcus parasanguinis 14137939_2 MVD (TSA)*	1.91
Non-AT4	-	*Streptococcus vestibularis DSM 5636T DSM (BHI)*	1.93
		*Streptococcus salivarius 0807M25049501 IBS (BHI/VRE)*	2.07
		*Streptococcus salivarius DSM 20560T DSM (TSA)*	2.07
Non-AT5	-	*Rothia mucilaginosa DSM 20446 DSM (BHI)*	1.80
		*Streptococcus vestibularis DSM 5636T DSM (TSA)*	2.05
Non-AT6	-	*Streptococcus vestibularis DSM 5636T DSM (BHI)*	1.94
		*Streptococcus parasanguinis CCUG 55521 CCUG (BHI)*	2.31
		*Streptococcus oralis DSM 20379 DSM (VRE/TSA)*	2.24
Non-AT7	-	*Streptococcus salivarius DSM 20560T DSM (BHI)*	2.30
		*Neisseria perflava DSM 18009T DSM (BHI)*	2.45
		*Streptococcus anginosus DSM 20563T DSM (VRE)*	2.20
		Escherichia coli ATCC 25922 CHB (TSA) *	2.29
Non-AT8	-	*Streptococcus salivarius 0807M25049501 IBS (BHI/VRE/TSA)*	2.17
Non-AT9	-	*Streptococcus oralis DSM 20395 DSM (BHI/VRE)*	2.20
		*Streptococcus oralis DSM 20627T DSM (TSA)*	2.26
Non-AT10	-	*Streptococcus salivarius 0807M25049501 IBS (BHI)*	2.11
		Staphylococcus aureus ssp aureus DSM 4910 DSM (TSA) *	2.51
Non-AT11	-	*Streptococcus salivarius DSM 20560T DSM (BHI)*	2.27
		*Neisseria perflava 1621 PGM (BHI)*	2.35
		Staphylococcus aureus ssp aureus DSM 4910 DSM (VRE) *	2.28
		*Lactobacillus plantarum DSM 1055 DSM (VRE/TSA)*	2.25
Non-AT12	-	*Neisseria flavescens C1 2 PGM (BHI)*	2.18
		*Neisseria perflava DSM 18009T DSM (BHI)*	2.49
		*Streptococcus oralis DSM 20379 DSM (TSA)*	2.48
Non-AT13	-	*Streptococcus vestibularis DSM 5636T DSM (BHI)*	2.13
		*Streptococcus gordonii DSM 6777T DSM (BHI)*	2.01
		*Staphylococcus aureus ATCC 33591 THL (VRE/TSA)*	2.12
Non-AT14	-	*Streptococcus vestibularis DSM 5636T DSM (BHI/TSA)*	2.08
		*Rothia dentocariosa DSM 43762T DSM (BHI)*	2.04
		*Streptococcus salivarius DSM 20560T DSM (VRE)*	1.80

**Table 2:** Identification results of all isolates identified in AT patients using MALDI-TOF MS

Patient name	Antibiotic	Best much in MALDI Biotyper database	Score
Antibotic treatment (AT)			
AT1	Azithromycin	*Streptococcus oralis DSM 20395 DSM (BHI)*	2.26
		*Streptococcus salivarius 0807M25049501 IBS (VRE/TSA)*	2.10
AT2	Amoxicillin	*Streptococcus parasanguinis CS 50_4 BRB (BHI)*	2.19
AT3	Piperacillin	*Staphylococcus haemolyticus Mb18803_2 (VRE)*	2.15
		Enterococcus faecium DSM 13589 DSM (VRE) *	2.29
AT4	Ciprofloxacin + metronidazole	*Streptococcus salivarius 0807M25049501 IBS (BHI)*	2.24
		*Staphylococcus hominis ssp novobiosepticus DSM 15614T DSM (AZB)*	2.42
AT5	Clindamycin	Escherichia coli DH5alpha BRL (BHI) *	2.26
		*Streptococcus oralis DSM 20627T DSM (BHI)*	2.08
		Enterococcus faecalis 20247_4 CHB (VRE/TSA) *	2.46
AT6	Clindamycin	Candida albicans ATCC 10231 THL (BHI) *	2.16
		*Streptococcus sanguinis DSM 20567T DSM (BHI)*	2.13
		Staphylococcus epidermidis 10547 CHB (VRE) *	2.30
		*Lactobacillus plantarum DSM 1055 DSM (TSA)*	2.30
AT7	Ciprofloxacin	*Streptococcus salivarius DSM 20560T DSM (BHI)*	2.36
		*Streptococcus parasanguinis 14137939_2 (BHI)*	2.18
		Enterococcus faecium DSM 17050 DSM (VRE) *	2.40
		*Streptococcus vestibularis DSM 5636T DSM (TSA)*	2.19
AT8	Clindamycin	Candida glabrata DSM 11950 DSM (BHI) *	2.29
		*Rothia dentocariosa B16575_bh8 IBS (BHI)*	2.16
		Staphylococcus epidermidis DSM 1798 (VRE)*	2.22
		*Lactobacillus plantarum DSM 1055 DSM (VRE)*	2.33
		*Streptococcus salivarius 0807M25049501 (TSA)*	2.06
AT9	Ciprofloxacin	*Streptococcus oralis DSM 20379 DSM (BHI/TSA)*	2.14
		*Lactobacillus paracasei ssp paracasei DSM 2649 (VRE)*	2.11
AT10	Piperacillin	*Streptococcus parasanguinis CS 50_4 BRB (BHI)*	2.05
		Candida albicans DSM 6569 DSM (VRE) *	2.15
		*Staphylococcus epidermidis 10547 CHB (TSA)*	2.08
AT11	Metronidazole	*Rothia mucilaginosa DSM 20445 DSM (BHI)*	2.23
AT12	Piperacillin	*Streptococcus parasanguinis CS 50_4 BRB (BHI)*	2.00
		Bacillus subtilis ssp subtilis DSM 10T DSM (BHI) *	1.60
		*Streptococcus salivarius 0807M25049501 IBS (VRE)*	2.21
		*Staphylococcus epidermidis 10547 CHB (TSA)*	2.23
AT13	Clindamycin + levofloxacin	*Streptococcus salivarius 0807M25049501 IBS (BHI)*	2.20
		*Streptococcus parasanguinis 14137939_2 MVD (TSA)*	2.29
AT14	Clindamycin	Enterococcus faecalis DSM 20409 DSM (BHI) *	2.54
AT15	Clindamycin	Candida albicans ATCC 10231 THL (BHI) *	2.04
		*Serratia marcescens DSM 12481 DSM (BHI)*	2.38
		Enterococcus faecalis DSM 20409 DSM (VRE) *	2.35
		*Enterococcus faecalis DSM 2570 DSM (TSA)*	2.34
AT16	Clindamycin	Candida dubliniensis 99 PSB (BHI) *	2.01
		Enterococcus faecium DSM 13589 DSM (VRE) *	2.29
		*Staphylococcus epidermidis DSM 1798 DSM (TSA)*	2.06
AT17	Clindamycin	*Neisseria flavescens C1 2 PGM (BHI)*	2.27
		*Micrococcus luteus IMET 11249 HKJ (TSA)*	2.10
AT18	Clindamycin	*Neisseria perflava DSM 18009T DSM (BHI)*	2.18
AT19	Cefotaxime	*-*	
AT20	Cefotaxime	*Rothia mucilaginosa BK2995_09 ERL (BHI)*	2.37
		*Streptococcus parasanguinis CS 50_4 BRB (BHI)*	2.44
		*Streptococcus parasanguinis 14137939_2 MVD (VRE)*	2.26
		Candida albicans DSM 6569 DSM (VRE) *	1.95
		*Staphylococcus epidermidis DSM 1798 DSM (TSA)*	2.13
AT21	Amoxicillin	Staphylococcus cohnii ssp urealyticus DSM 6718T(BHI) *	2.13
AT22	Clindamycin	*Neisseria mucosa 1591 PGM (BHI)*	2.12
		*Staphylococcus hominis ssp novobiosepticus DSM 15614T DSM (BHI/TSA)*	2.43
		*Staphylococcus epidermidis ATCC 14990T THL (VRE)*	2.16
AT23	Clindamycin	*Rothia dentocariosa RV_BA1_032010_D LBK (BHI)*	2.35
		*Staphylococcus hominis 18 ESL (VRE)*	2.17
		*Staphylococcus hominis ssp novobiosepticus DSM 15614T DSM (TSA)*	2.15
AT24	Clindamycin	*Neisseria flavescens C1 2 PGM (BHI)*	2.24
		*Staphylococcus epidermidis ATCC 12228 THL (VRE)*	1.83
		Enterococcus faecalis 20247_4 CHB (TSA) *	1.80

The only one - *B. subtilis* (AT12) was found to be below 1.7 (log (score) = 1.6). Moreover, it is necessary to underline that *S. aureus* and *S. pneumoniae* were noticed only in non-AT group, whereas *E. faecalis, E. faecium*, *S. epidermidis*, *B. subtilis*, *S.cohnii* and yeasts *C. albicans*, *C. glabrata*, and *C. dubliniensis* were found only in AT group. Based on the shown date, it can be noticed that the identification for *S. salivarius* cultivated on various medium was similar (score value for non-AT4 was 2.07), (Table 1). However, the protein profile of the identified bacteria differed (Fig. 1B). The comparison of the protein profile of *Streptococcus salivarius* based on the culture medium was presented in Fig. 1B. In the case of the non-AT patients, the mass spectra show that the signals at *m/z* = 4451 and *m/z* = 5968 are similar in BHI, VRE, and TSA. Moreover, it can be observed that the differences in intensities of generated signals depend on the culture medium used. However, the signal 2762 *m/z* was recorded only in the universal media (BHI, TSA). In contrast, this signal disappeared in the case of the VRE medium, and the new signal was registered at 2984 *m/z*. Moreover, the signal *m/z*= 7888 was observed in the protein profile of *S. salivarius* identified on the BHI and VRE growing media in the non- and treated group. It is notable that some signals are present only in one mass spectra of non-AT4 in the case of each medium: 9030 and 10268 *m/z* in BHI; 9443 *m/z* in VRE and 10840 *m/z* in TSA. Based on the mass spectra of *S. salivarius* in the patient group treated with antibiotics (AT13 and AT1), the common signals (*m/z* = 2984; *m/z* = 4451; *m/z* = 5968) can be noticed. Furthermore, the *m/z* = 10068 was noticed in the universal media (BHI and TSA). However, the differences in the protein profile of the studied bacteria strain were also recorded. The signals at 9090 *m/z* were observed in BHI and at 9444 *m/z* in the VRE growing medium.

It is notable that the relative intensities and the noise level of the signals of the registered protein profiles depended on the growing employed media. Hence, the use of the selective culture media that contains various components, including antibiotics, salts, and pH indicators can be the only limitation associated with the use of the mass spectrometric techniques. Some components, such as salts, are well-known inhibitors for the mass spectrometry, and various media can induce changes in the bacterial protein expression (Metwally et al. 2015). Therefore, it is supposed that the disappearance of some signals in the mass spectra recorded after the use of the VRE medium of *S. salivarius* can be correlated with the salt mixture present in this selective culture medium. Karamonová and co-workers (Karamonová et al. 2013) established the optimal cultivation media for the identification of *Cronobacter sakazakii* bio groups using the MALDI-TOF MS. They studied the universal growth media, such as TSA, BHA, Blood Agar Base (Blood Agar Base with sheep blood (5%), BA), and selective cultivation medium *Enterobacter sakazakii* Isolation Agar (ESIA). It was observed that the intensity of the recorded mass spectra was lower in the case of the ESIA medium than in the universal media (TSA, BHA, BA). It was suggested that the unsatisfied intensity of the protein profile of *Cronobacter sakazakii* CB03 can be caused by the deficient composition of the selective medium rather than the universal media and the presence of specific (selective) substances (sodium desoxycholate, sodium chloride, and crystal violet) as inhibitors of growing microbial competitors. Finally, the TSA medium was chosen to further analysis by the MALDI-TOF MS (Karamonová et al. 2013).

Additionally, the mass spectra of *Streptococcus vestibularis* between the non-antibiotic treatment (non-AT5) and the antibiotic treatment (AT7) patients were also compared (Fig. 1C). The common signals at 3944, 4452, and 5968; 6755, 7888 *m/z* (Fig. 1C) and at 2984 *m/z* (Fig. 1C-Z2), 5135; 5188 *m/z* (Fig. 1C-Z4), and 6311*m/z* (Fig. 1C-Z6) were found in both studied groups. Moreover, the signals at 2731, 2743, 2762, 2778, 2800, 2816 m/z (Fig. 1C-Z1), at 2907 *m/z* (Fig. 1C-Z2), at 4723; 4788 *m/z* (Fig. 1C-Z3), and at 6139 *m/z* (Fig. 1C-Z6) were characteristics only for the non-AT patients. However, in the protein profile of *S. vestibularis* (AT7), the non-common signals *m/z* 4923 (Fig. 1C-Z3), *m/z* 5363, 5391, and 5557 (Fig. 1C-Z5) were also observed. According to the UNIPROT database, the common signals registered at 4452 *m/z* indicated the 50S ribosomal protein L36 (structural constituent of ribosome) (Maeder and Draper 2005) while those registered at 5968 *m/z* were found to be responsible for the defense response to bacterium (Bacteriocin-type signal sequence) (Wescombe et al. 2009). Remarkably, the disappearance of the signal at *m/z* 4723 in the AT group, responsible for the DNA binding and transpose activity can be associated with the mechanism of antibiotics, in this case, ciprofloxacin (it inhibits the DNA replication) (LeBel 1988).

**Fig. 2:**
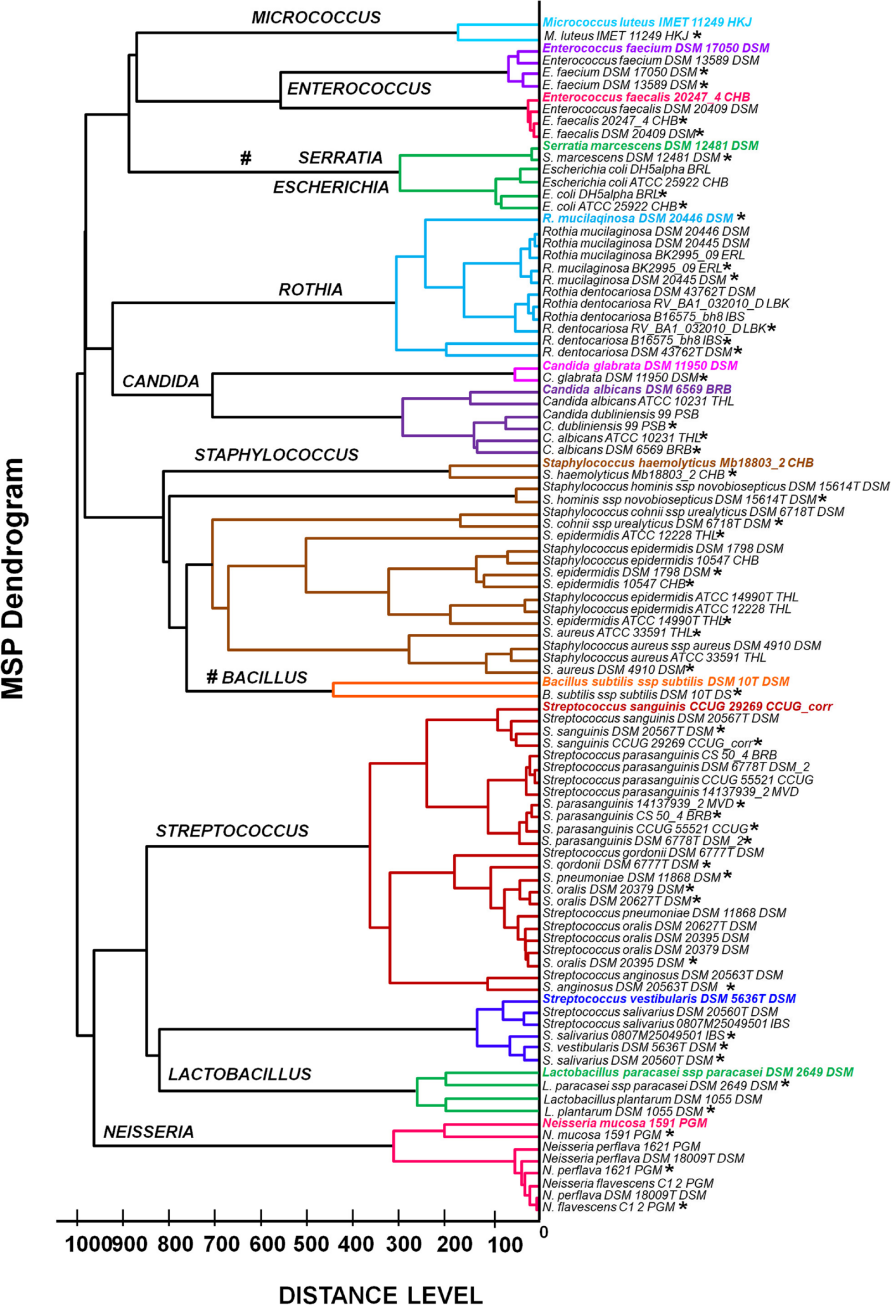
The phyloproteomic tree of all identified isolates based on the obtained  MSP identification via MALDI Biotyper platform

In the next step, the recorded mass spectra for all the isolates were matched to the reference spectra (MSPs), and the phyloproteomic tree was created. Figure 2 represents the hierarchical clustering of the identified isolates and correlation to the reference species (from the MALDI database). Based on the MSP dendrogram (Fig. 2), 11 main clusters indicating genus (*Micrococcus, Enterococcus, Serratia, Escherichia, Rothia, Candida, Staphylococcus, Bacillus, Streptococcus, Lactobacillus,* and *Neisseria*) were noticed. The relationship between the identified microorganisms and the reference strains was found. The showing longest distance level correlation between the bacteria strain was observed in the cluster belonging to the *Staphylococcus* genus. Moreover, the cluster describing the *Enterococcus* genus indicates the shortest distance among the identified species. According to the dendrogram, a close relationship between the *Serratia* and *Escherichia* genus can be noticed. Moreover, the *Bacillus* genus was also included in the phyloproteomic tree (Fig. 2), and (marked by #), despite the low identification level using the MALDI-TOF MS technique (1.7 >). Furthermore, a close relationship between *Streptococcus salivarius* and *Streptococcus vestibularis* was noticed. According to the research previously published by our group (Złoch et al. 2020b), the problem of distinguishing those species could be overcome by using the MALDI-TOF MS to create protein and lipid profiles.

Considering the manufacturer’s guidelines, the low score value of 1.6 (Table 2), only for *Bacillus subtilis* was obtained while the standard method for the bacteria identification (16S rRNA gene sequencing) was performed. Furthermore, to confirm the accuracy of the MALDI results for all the identified 11 genus of bacteria, the one species from each cluster was selected (*Neisseria perflava*, *Lactobacillus plantarum*, *Streptococcus salivarius*, *Staphylococcus aureus*, *Staphylococcus epidermidis,* and *Enterococcus faecalis*). In addition, the *Streptococcus pneumoniae* as a serious pathogen and *Staphylococcus cohnii* showing the high level of the antibiotic resistance were also chosen. However, the genus with the low abundance of percentage in the identification including *Candida*, *Rothia*, *Escherichia*, *Serratia*, and *Micrococcus* was considered in the PCR analysis.

Then, the results received by the MALDI-TOF MS (score > 2.00) were correlated with the 16S rRNA gene sequencing method (excluding the *Bacillus subtilis*). However, the low identification level was verified in the PCR analysis. On the basis of the data from the PCR assay, the value of identification was over 99.5% for all the studied bacteria species. Moreover, the following accession numbers were given to the bacteria: *B. subtilis* (MZ336018); *N. perflava* (MZ191898); *Lactiplantibacillus plantarum* (the previous form *Lactobacillus plantarum*) (A taxonomic note on the genus *Lactobacillus*) (MZ411566); *S. salivarius* (MZ191906); *S. aureus* (MZ191908); *S. epidermidis* (MZ411533); *E. faecalis* (MZ191905); *S. pseudopneumoniae* (MZ191882), and *S. cohnii* (MZ191897).

Based on the PCR method, in one case, only the identification compared to the MALDI- TOF MS was slightly different. The protein profile of *S. pneumoniae* was identified as *S. pseudopneumoniae* using the 16S rRNA gene sequencing method. Lucia Gonzales–Siles et al. studied the genomic markers for the differentiation and identification of both *Streptococcus* species. The presence of these unique markers was confirmed by the PCR with reference strains and clinical isolates (Gonzales-Siles et al. 2020).

**Fig. 3:**
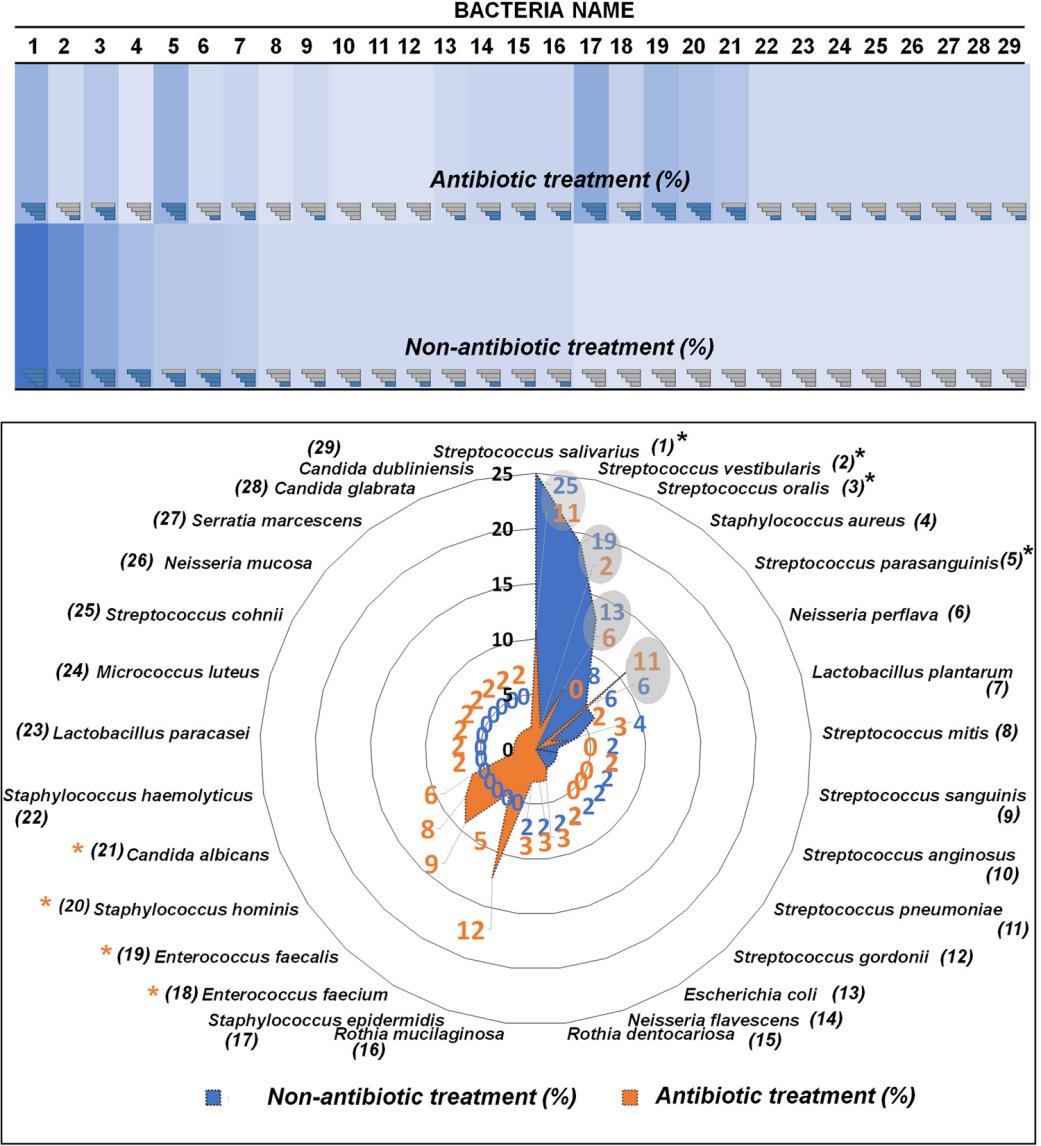
The heat map (up) representing the abundance of identified isolates and radar chat (down) showing the % distribution predominance of all identified microorganism species in non-AT and AT group

Summarily, 29 already identified species were represented as predominant species in both non-AT and AT salivary sample groups (Fig. 3). The diversity in the salivary bacteria in the AT group vs the non-AT was observed. Figure 3 (up) illustrates the heat map representing the abundance of all identified species of microorganisms (%).

Moreover, based on the created heat map, the % distribution predominance of all identified microorganism species was performed and shown as a form of the radar chart (Fig. 3 down).

Regarding the investigated radar chat, the differences between the salivary microbiota of non-AT and AT patients were found. It can be observed that the *Streptococcus salivarius* (25%) and *Streptococcus vestibularis* (19%) dominated in the non-antibiotic treatment patients. Another predominant bacteria species in patients with the normal salivary microbiome were *Streptococcus oralis* and *Staphylococcus aureus.*

The blue area of the web chart (Fig. 3 down) shows that in the non-AT group, the 16 species of bacteria (from *S. salivarius* to *R. mucilaginosa*) were identified. Compositionally, the most abundant microorganism present in the AT patients were *Streptococcus salivarius*(11%) *Streptococcus parasanguinis* (11%), *Staphylococcus epidermidis* (12%), *Enterococcus faecalis* (9%), *Staphylococcus hominis* (8%), *and Candida albicans* (6%).

The orange color indicates that the saliva of patients under the antibiotic therapy was more bacterially rich than the non-AT group. It was also observed that pathogenic microorganisms dominated in the group. It can be assumed that the type of the antibiotic treatment influenced the salivary bacteria composition in the AT patients. In comparison with patients with normal (physiological) salivary microbiota, more diversity of microorganism and more abundance of pathogenic bacteria can be noticed in the AT group, which can be associated with stress conditions under the antibiotic treatment.

**Fig. 4:**
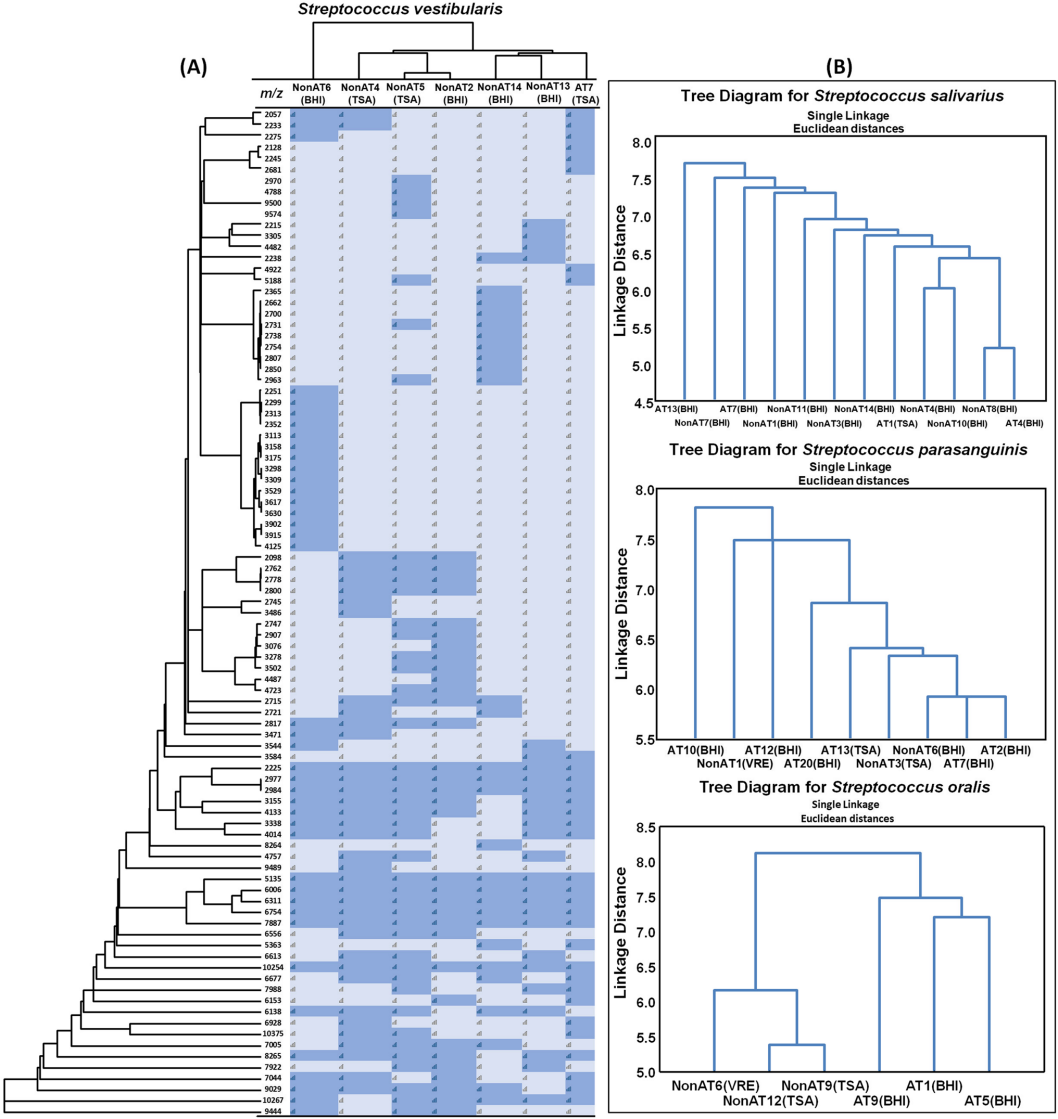
Heat map (**A**) and formed clusters (vertical left) illustrating the differences between registered common and characteristic signals in both groups; hierarchical clustering distinguishing non-AT and AT for *S. vestibularis* (**A**, up), for *S. salivaris*, *S. parasanguinis*, and *S. oralis* (**B**)

**Fig. 5:**
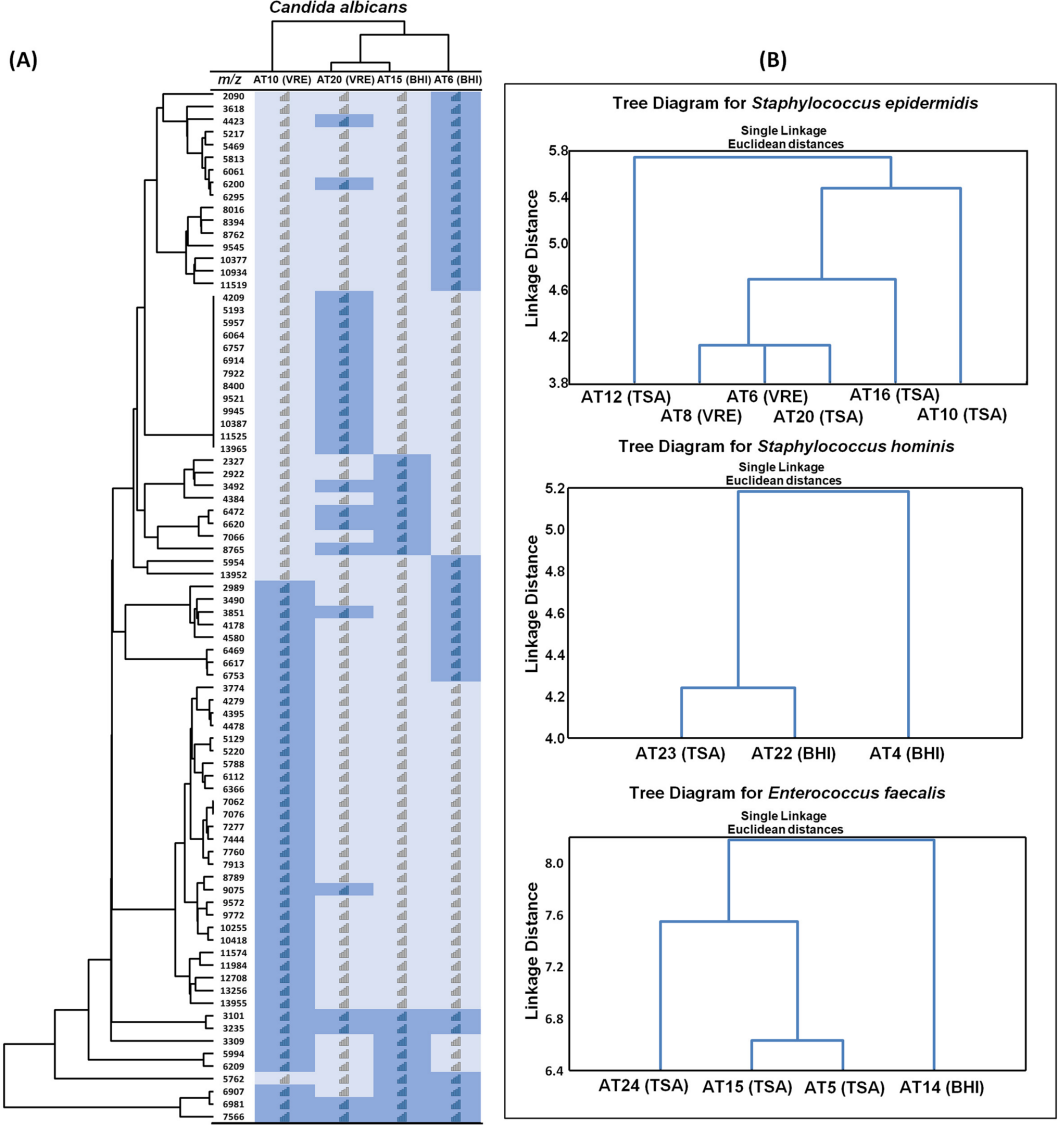
Heat map (**A**) and formed clusters (vertical left) illustrating the differences between registered common and characteristic signals only in AT; hierarchical clustering distinguishing different AT for *C. albicans* (**A**, up), for *S. epidermidis*, *S. hominis*, and *E. faecalis* (**B**)

The correlation between the identified pathogen and the antibiotic was shown in the cluster analysis (Figs. 4 and 5). For further discussion, the most predominant species identified in both non-AT and AT samples were taken into consideration.

The analysis of the intraspecific proteomic variation within distinct microbial species derived from the saliva of patients treated and untreated with antibiotics revealed the impact of the treatment undertaken, associated microbiota, and applied culture conditions on the generated protein profiles (Figs. 4 and 5). Regarding species that occurred in both patients group, obtained results indicated the influence of the antibiotic used and the type of the culture medium on the variation in the proteomic composition of the bacteria (Fig. 4). As the example of the species *S. vestibular*is is shown (Fig. 4A), such differences cover a wide range of *m/z* with various frequencies. In that particular case, the most different protein pattern was noted for the strains isolated from the patient non-AT6 and AT7 and can be attributed to the synergic effect of the culture medium composition (TSA vs*.* BHI) and antibiotic treatment (ciprofloxacin). A similar effect was observed for *S. oralis* (Fig. 4B), where isolates collected from the AT patients and cultured on the BHI comprised a distinct group. Moreover, *S. oralis* isolated from the non-AT also revealed a clear grouping according to the culture medium—VRE vs. TSA. Although all *S.oralis* from the AT patients were isolated using BHI, their proteome differentiation was much higher than that of isolates detected among the non-AT, which can be associated with a different antibiotic treatment—ciprofloxacin (AT9), azithromycin (AT1), and clindamycin (AT5). Regarding the other two strains found in both patients group—*S. salivarius* and *S. parasanguinis*—the comparative analysis revealed the highest proteomic variations; however, grouping according to drug-treated and untreated patients was not as evident as in the former cases. Nonetheless, *S. parasanguinis* strains isolated from the AT patients were placed into different clusters according to the type of antibiotic used, where strains from piperacillin-treated patients (AT10 and 12) were much more similar to each other than those from ciprofloxacin, amoxicillin, and clindamycin with levofloxacin (Fig. 4B). Additionally, great diversity noted for *S. parasanguinis* can be related to various microbiota found in the saliva samples. Indeed, strains isolated from samples accompanied by the presence of such microorganisms as *Candida albicans* (AT10 and 20) or *Bacillus subtilis* (AT12) demonstrated a more unique proteins pattern than those comprised of streptococci only (AT2 or non-AT6). A similar phenomenon was noted for *S. salivarius*, where the strains showing extremely different protein profiles came from samples significantly different in the species composition—non-AT7 (additionally *N. perflava*, *S. anginosus*, *E. coli*) vs. non-AT8 (*S. salivarius* only)—or the antibiotic used—AT13 (clindamycin with levofloxacin) vs AT4 (clindamycin with metronidazole). All in all, comparing species derived from the saliva of treated and untreated patients, the impact on the proteomic diversification increases as follows: the type of the antibiotic used>coexistent microbiota>culture medium type.

Similarly, a comparison of the proteomic diversity among microbial species that occurred only in the AT patients was performed (Fig. 5). The effect of the culture medium used was noted for *C. albicans* (Fig. 5A) along with *E. faecalis* strains (Fig. 5B). Interestingly, in the case of *E. faecalis*, the strain isolated as a single microorganism from patient AT14 demonstrated a far more different proteins pattern compared to the rest of the strains which were isolated from samples occupied by pathogenic microorganisms like *C. albicans*, *S. marcescens*, *N. flavescens* or *E. coli*. In turn, the proteomic intraspecific diversification of the staphylococci (*S. hominis* and *S. epidermidis*) most likely resulted from the type of antibiotic treatment undertaken—ciprofloxacin with levofloxacin vs. clindamycin in the case of former and piperacillin vs. clindamycin or cefotaxime in the latter one. Moreover, among *S. epidermidis* strains the most distinct proteins profile was demonstrated by the isolate AT12—the only one that derived from the sample in which the presence of *Candida* spp. was not observed.

## Discussion

Intraspecific differences in microbial protein profiles depending on culture media compositions were recognized as significant since 50% of the peaks of all bacteria are non-ribosomal proteins, which are more or less metabolic status dependent (Mellmann et al. 2008). It was proved in the work of Złoch et al. (Złoch et al. 2020a) where changing the culture conditions significantly influenced the differentiation of *S. aureus* strains based on their protein patterns. As the authors pointed out, it may result from the induction of some new metabolic pathways in bacteria leading to the appearance of more discriminant signals.

According to previous reports (Monedeiro et al. 2021; Szultka-Młyńska et al. 2021) the culture media could be useful for the separation of each bacterial strains from the sample obtained from the hospital. In this context, the present research have also used different media to select and identify the individual strain. Moreover, it has been investigated how the composition of the used media can influence the isolation and identification of each bacteria in order to establish an optimal media in this way regarding the biomedical approach. In general, the identification of microorganisms by MALDI is considered culture independent, since most of the proteins present in bacterial cells are ribosomal proteins (about 50% or more), so that reliable classification of bacteria for most genera and species is certain regardless of the culture media used. Nevertheless, in addition to ribosomal proteins, the bacterial extracts studied also contain other non-ribosomal proteins that are more or less metabolism dependent. Such proteins can affect the identification result primarily in the case of bacteria belonging to groups of closely related species (e.g., *Bacillus subtilis* or cereus group, *S. salivarius* group or *S. mitis*/*oralis*) leading to misidentification. Knowing that in the case of closely related species, the genomic and proteomic differences are very small, even slight variation in the culturing conditions may matter.

Nevertheless, the results of our studies showed that the impact of the culture medium type on the intra-specific variation of the proteomes was lower than the effect of the antibiotic treatment and the presence of the co-exist microbiota. It was shown that the interaction that occurred between microbes can alter the expression of their membrane proteins. Such a phenomenon was noted in the work of Kumar and Ting, where amounts of seven classes of proteins on the *S. aureus* surface were elevated upon coculturing with *P. aeruginosa* (Kumar and Ting 2015). Found proteomic changes included proteins related to host-microbe interactions such as virulence, adhesion, and resistance, which explains the increased fatality of infections with the simultaneous presence of *Staphylococcus aureus* and *Pseudomonas aeruginosa* compared to the colonization of the individual bacterial species (Fazli et al. 2009). A similar phenomenon was observed in our studies where for instance *E. faecalis* and *S. salivarius* that co-existed with other microbial species demonstrated distinct protein patterns from the ones isolated as a monoculture. Since most of the accompanying species represented pathogenic microorganisms, the detected intra-specific proteome variation of the mentioned bacterial species may be partially explained by the horizontal transfer of the genes related to virulence factors. Bacterial genomes frequently contain a significant amount of foreign DNA, which is DNA that originated from another organism and was inserted into the genome of a bacterium (Ochman et al. 2000). The DNA mobilized into the host bacterium is referred to as mobile genetic elements (MGEs), which have a huge impact on the shape of the bacterial genomes and promote intra-specific variability (Heuer and Smalla 2007). *Enterococcus faecalis* harbors a pathogenicity island containing several virulence factors and is known for its fast adaptation to the clinical environment by the acquisition of antibiotic resistance and pathogenicity traits generating it the third leading cause of hospital-associated infections (Laverde Gomez et al. 2011). Although the horizontal gene transfers occur more frequently between closely related bacteria, they also occur among distantly related species. Nevertheless, horizontally acquired (or lost) genes can also contribute to ecological adaptation, and they are likely to be major drivers of niche differentiation among bacteria (Smillie et al. 2011). Moreover, different habitats can be expected to support different levels of intra-species diversity and to be subject to distinct selection pressures (Ellegaard and Engel 2016). Considering clinical specimens, antibiotics demonstrate highly selective pressure on the bacteria populations. Besides, causing the shifts in the species composition, it was proved that antibiotics (at certain concentrations which depend on their class) are also responsible for the high phenotypic variation even at a single bacterial population level (Lee et al. 2018). Indeed, the presence of the antibiotic demonstrated the highest impact on the proteomic intra-specific diversity of the investigated salivary microorganisms, including *Candida* spp. Moreover, such an impact depends on the type of the antibiotic used and tested microorganisms.

Our results indicated significant differences in the saliva microbiota between non-antibiotic treatment and antibiotic treatment patients. We noticed the dominated and characteristic microorganisms in the non-AT (*Streptococcus salivarius* (25%) and *Streptococcus vestibularis* (19%), *Streptococcus oralis* (13%) and *Streptococcus parasanguinis* (6%)) and in the AT (*Streptococcus salivarius* (11%), *Streptococcus parasanguinis* (11%), *Staphylococcus epidermidis* (12%), *Enterococcus faecalis* (9%), *Staphylococcus hominis* (8%), and *Candida albicans* (6%)) groups. The salivary microbiota of antibiotic-treated patients was characterized by a more bacteria variety; the appearance of the *Candida albicans* species was noticed only in the AT patient indicating a negative impact of the antibiotic administration on the patient microbiota. Moreover, the proteomic analysis showed the influence of the antibiotic therapy, composition, and abundance of saliva microbiota and used growth medium on the recorded protein profiles. Remarkably, the MALDI-TOF MS analysis represents a promising method for a large-scale, less labor intensive, rapid, and cost-effective record of reproducible molecular profiles of microorganisms, particularly the salivary bacteria. It is notable that the proposed approach enables the early administration of the specie-specific antimicrobial therapy in the patients. Therefore, our data can allow a medical diagnosis to be confirmed and they may also enable us to monitor the treatment of diseases and develop drugs for individual patients. Moreover, the presented study can be the base to develop methods enabling a faster diagnosis and the detection of disease changes at the cellular level before clinical changes occur. To summarize, the obtained results suggest that tracking proteomic intra-specific variation within microorganisms may be a promising tool for the future use to examine the effectiveness of the applied antibiotic curation and to check whether one deals with mono- or polymicrobial cultures including the presence of the other pathogenic species.

## Data Availability

Authors can confirm that all relevant data are included in the article and/or its supplementary information files.
